# Supplementary material for: Quality of Life and Physical Activity of Persons with Spinal Cord Injury
Source: Int J Environ Res Public Health. 2021 Aug 30;18(17):9148. doi: 10.3390/ijerph18179148 (PMC8430911; doi:10.3390/ijerph18179148)
Supplement: Supplementary file 1 [file ijerph-18-09148-s001.zip › ijerph-1315207-supplementary.pdf]

Supplementary file 1

Table S1. List of dependent variables by areas of the quality of life.

Area	Dependent variable	Content of the variable
**Material prosperity**	Satisfaction with income	Financial situation (income, taxes)
	The ability to take care of oneself and one's family	Employment (work, status)
	Ability to work	A sense of ability to work
	Obstacles– Employment	Obstacles to employment due to the secondary conditions
**Physical wellbeing**	Health	Health (functioning, symptoms, capacity), medical care
	Obstacles– Daily tasks	Obstacles to daily tasks due to secondary conditions
	Capacities – Daily tasks	Everyday activities (ordinary skills, self-care, mobility)
	Leisure activities	The way of spending one's leisure time (recreation, hobbies)
**Personal development**	Education	Desire for education (achievements, status)
	Obstacles – Education	Obstacles to education due to secondary conditions
	Concentration	Ability to focus on learning, work
	Overcoming obstacles	The methods used to overcome obstacles
	Personal competence	Satisfaction with personal qualifications (cognitive, social, practical)
	Success	Experiencing success (accomplishments and achievements in life)
	Productivity	Experiencing productivity in life
**Emotional well-being**	Life satisfaction	Experiencing satisfaction (feelings of enjoyment and a good mood), stress reduction (feelings of predictability and control)
	Optimism, hope, Acceptance of reality	Positive acceptance of life
	Meaning of life	A life that is worth living and meaningful
	Self-acceptance	Self-image (identity, self-esteem, having a good opinion about oneself)
	Slow progress	Acceptance of slow progress
	Religiosity	Finding help, peace, and certainty in believing in God
	Safety	A sense of security
**Self-determination**	Autonomy	The ability to independently manage a new way of life
	Personal control	A sense of personal control and autonomy (independence)
	Respecting decisions, values	Respect for an individual's goals and personal values (desires, expectations, opinions)
	Personal choices	Possibility of independent choices (where to go, with whom, how to spend money, etc.)
	Independent decision making	Others making decisions about the individual's life
**Interpersonal relationships**	Good relations with friends	Interactions (social networks, social contacts) that are relaxed and positive with friends, roommates, neighbors, and acquaintances
	Leisure time with friends	Spending leisure time with friends, neighbors, and others who exercise
	Getting along with one's family	Compassionate, intimate, friendly relationships with family members

	Leisure time with family	Spending leisure time with family members
	Care and support in the family	Support (emotional, physical, and financial, receiving some feedback)
**Social inclusion**	Activities in the family	Active involvement in family activities
	Activity in one’s place of residence	Integration and participation in one’s place of residence
	Feeling of importance	A sense of importance in one's community
	Role in the community	Role in one’s community (as co-worker, volunteer, etc.)
	Social help and support	The potential for social support (support networks, assistance)
	Accessibility and support	Satisfaction with access to, support and assistance of health services
**Rights**	Equality	Experiencing and enforcing the rights to equality and non-discrimination
	Privacy	The possibility of privacy, retreat
	Dignity	Experiencing dignity
	Environmental barriers	Accessibility, removal of environmental barriers
	Timeliness of processes	Justice and timeliness in judicial, medical and other processes
